# Food consumed outside the home in Brazil according to places of purchase

**DOI:** 10.1590/S1518-8787.2017051006750

**Published:** 2017-02-21

**Authors:** Ilana Nogueira Bezerra, Tyciane Maria Vieira Moreira, Jessica Brito Cavalcante, Amanda de Moura Souza, Rosely Sichieri

**Affiliations:** I Mestrado Acadêmico em Nutrição e Saúde. Centro de Ciências da Saúde. Universidade Estadual do Ceará. Fortaleza, CE, Brasil; II Centro de Ciências da Saúde. Universidade de Fortaleza. Fortaleza, CE, Brasil; III Departamento de Epidemiologia e Bioestatística. Instituto de Estudos em Saúde Coletiva. Universidade Federal do Rio de Janeiro. Rio de Janeiro, RJ, Brasil; IV Departamento de Epidemiologia. Instituto de Medicina Social. Universidade do Estado do Rio de Janeiro. Rio de Janeiro, RJ, Brasil

**Keywords:** Food Habits, Food Consumption, Food Services, Street Food, Collective Feeding, Consumer Behavior, Socioeconomic Factors, Diet Surveys

## Abstract

**OBJECTIVE:**

This study aims to describe the places of purchase of food consumed outside the home, characterize consumers according to the places of consumption, and identify the food purchased by place of consumption in Brazil.

**METHODS:**

We have used data from the *Pesquisa de Orçamento Familiar* (Household Budget Survey) of 2008-2009 with a sample of 152,895 subjects over 10 years of age. The purchase of food outside the home was collected from the records of all expenditures made in seven days. The places of purchase were grouped according to their characteristics: supermarket, bakery, street food, restaurant, snack bar, fruit shop, and other places. The types of food were grouped into nine categories, considering the nutritional aspects and the marketing characteristics of the item. We have estimated the frequency of purchase in the seven groups of places in Brazil and according to gender and type of food purchased per place. We have calculated the average age, income and years of education, as well as the *per capita* expenditure according to places of purchase of food consumed outside the home.

**RESULTS:**

The purchase of food outside the home was reported by 41.2% of the subjects, being it greater among men than women (44% *versus* 38.5%). Adults had a higher frequency (46%) than teenagers (37.7%) and older adults (24.2%). The highest frequency of places of purchase were snack bar (16.9%) and restaurant (16.4%), while the fruit shop (1.2%) presented the lowest frequency. Sweets, snack chips and soft drinks were the most purchased items in most places. Average expenditure was higher for restaurant (R$33.20) and lower for fruit shop (R$4.10) and street food (R$5.00).

**CONCLUSIONS:**

The highest percentage of food consumed outside the home comes from snack bars and restaurants, pointing to important places for the development of public policies focused on promoting healthy eating.

## RESUMO

**OBJETIVO:**

Descrever os locais de aquisição dos alimentos consumidos fora do lar, caracterizar os consumidores de acordo com os locais de consumo e identificar os alimentos adquiridos por local de consumo no Brasil.

**MÉTODOS:**

Utilizaram-se dados da Pesquisa de Orçamento Familiar 2008-2009 em uma amostra de 152.895 indivíduos acima de 10 anos. A aquisição de alimentos para consumo fora do lar foi coletada por registros de todos os gastos realizados no período de sete dias. Os locais de aquisição de alimentos foram agrupados de acordo com suas características: supermercado, padaria, comida de rua, restaurante, lanchonete, frutaria e outros. Os tipos de alimentos adquiridos foram alocados em nove categorias de alimentos, considerando os aspectos nutricionais e as características de comercialização do item. Estimou-se a frequência de aquisição de alimentos nos sete grupos de locais no Brasil e por sexo e o tipo de alimento adquirido por local. Calculou-se a média de idade, de renda e de anos de escolaridade, bem como da despesa *per capita* segundo locais de aquisição de alimentos consumidos fora do lar.

**RESULTADOS:**

A aquisição de alimentos fora do lar foi reportada por 41,2% dos indivíduos, sendo maior entre os homens do que nas mulheres (44% *versus* 38,5%). Os adultos apresentaram maior frequência de aquisição (46%) do que os adolescentes (37,7%) e os idosos (24,2%). Os locais com maiores frequências de consumo de alimentos fora do lar foram lanchonete (16,9%) e restaurante (16,4%), enquanto frutaria (1,2%) apresentou a menor frequência. Doces, salgadinhos e refrigerante foram os alimentos mais adquiridos na maioria dos locais. Os gastos médios com alimentos foram maiores para restaurante (R$33,20) e menores para frutaria (R$4,10) e comida de rua (R$5,00).

**CONCLUSÕES:**

O maior percentual de consumo de alimentos fora do lar é proveniente de lanchonete e restaurante, apontando importantes locais para o desenvolvimento de políticas públicas com foco na promoção da alimentação saudável.

## INTRODUCTION

The pattern of food consumption of the population in several countries has been changing over the years^14^ ^,^ ^15^. In Brazil, although most meals are still eaten in the home (68.9% of expenditures on food in 2008-2009), food consumed outside the home has increased as we can see by the growth of the percentage of expenditure on it^a^. This demand for ready meals has favored the increase in the number of food establishments and has diversified their services, with emphasis on the growth in the number of restaurants, convenience stores, fast food chains, bakeries, among others.

Among the places of purchase of food, we can highlight fast food chains, which have grown and improved their menu^1^. Generally, these chains offer food with low nutritional value^9^, which contributes to the development of obesity^4^. In addition to modern fast food chains, the segment of street food is important in the sector of food outside the home^7^, and we can observe positive association between subjects who eat on the street and higher rates of prevalence of obesity and hypertension^6^.

In Brazil, buffet restaurants have both healthy options and food with low nutritional value, which can help to increase the prevalence of overweight and obesity^b^. In contrast to places that promote weight gain, the literature shows lower levels of obesity for supermarkets^13^, possibly because of the greater supply of healthy food^11^.

Spending with food eaten outside the home and the number of establishments that offer ready meals have increased in Brazil, as well as the prevalence of overweight. However, Brazilian studies focusing on the places of purchase of food consumed outside the home are limited. Costa et al.^8^ have investigated the contribution of places that sell food in the diet consumed in the home, observing the nature, purpose, and degree of processing of the food purchased. However, no places of purchase of food for consumption outside the home were assessed. The objective of this study was to describe these places, characterize consumers according to the places of consumption, and identify the food purchased in Brazil.

## METHODS

For this study, we used data from the *Pesquisa de Orçamentos Familiares* (POF – Household Budget Survey) of 2008-2009, carried out by the Brazilian Institute of Geography and Statistics (IBGE). The POF assesses the household budget of families (consumption, expenditure, and income)^a^.

A previous publication details the sample of the POF^a^. In summary, the POF of 2008-2009 was carried out in a probabilistic sample of 55,970 households, using a complex sampling plan based on a set of census sectors named “master sample”. The census sectors were geographically and statistically stratified and were selected in two stages. In the first stage, a quantitative amount of the census sectors were selected from the master sample with probability proportional to the number of households in each stratum and, in the second stage, the households were selected by simple random sampling. In total, 4,696 sectors of the master sample were selected and the interviews were carried out in 55,970 households, amounting to 190,159 residents^a^.

For this study, no children under 10 years of age were included (n = 31,977), since these children are not considered as budget units and their expenses are not individually recorded. Pregnant (n = 1,698) and lactating women (n = 3,589) were also excluded as eating habits often change, as well as what potentially influence their choice of places and the types of food consumed in these periods. The final sample amounted to 152,895 subjects.

The information regarding food consumption outside the home was obtained from a notebook filled by all members of the household who were 10 years of age or older. For a week, information on the product acquired, the form of acquisition, the amount of the expenditure in Real, and place of purchase were recorded. The record of the purchase could be made both in detail (item by item, as the food was acquired at each place) and resumed (a single record with the total spent during the week per place of purchase)^a^.

Household income was estimated by both monetary (cash, check and credit card, installments) and non-monetary income (donation, exchange, business, own production, fishing, and hunting) of the members of the family. The *per capita* household income was calculated by dividing household income by the number of residents.

The years of study of each resident were estimated by the IBGE from the last grade successfully completed. For subjects who have non-serial grades, the years of study were estimated as four years^a^.

The records of the spending with food outside the home amounted to 306 places of purchase of food, which were classified into eight groups according to the similarities in the structure of sale and supply, following definitions already used in other studies: 1) supermarket: stores that stock meat, wheat bread, fruits, vegetables and milk for retail or wholesale sales^20^; 2) bakery: establishments that offer quick meals and sandwiches, served at the counter or table^18^; 3) street food: food sold by street vendors, food and beverages ready for consumption, prepared or sold on the streets and other similar public places for immediate consumption or for later on without any preparation or processing^c^; 4) snack bar: establishments that predominantly sell preprocessed food and preparations according to a request^18^; 5) restaurant: establishments that offer full table service, waiters, and menus^18^ – the buffet restaurant was also included in this category; 6) fruit shop: places that mainly sell fruits and vegetables; 7) schools; and 8) other places: places that do not sell food as their main activity. For this work, the school group was not analyzed as it is a place subjected to strategies that regulate the supply of food, such as, for example, the *Programa Nacional de Alimentação Escolar* (PNAE – National Program of School Meals)^d^. We describe what we have included in each group in Table 1.

**Table 1:** Description and frequency of places (% and 95% confidence interval) of purchase of food consumed outside the home, according to gender. Brazil, 2008-2009.

Place	Description	Frequency		Gender			

				Male		Female	
		
		%	95%CI	%	95%CI	%	95%CI
Supermarket	Supermarket; hypermarket; food warehouse; market; grocery store; wholesale.	4.0	3.9–4.2	3.8	3.6–4.0	4.3	4.0–4.5
Bakery	Bakery; bakeshop; patisserie; bakehouse; delicatessen; coffee shop; confectionery store, rotisserie store.	4.1	3.9–4.3	4.3	4.1–4.6	4.0	3.7–4.2
Street food	Hawker; beach seller; trailer or kiosk; food cart.	4.6	4.4–4.8	4.3	4.1–4.5	4.8	4.6–5.1
Snack bar	Snack bar; ice cream parlor; pizzeria; confectionery store.	16.9	16.6–17.3	16.8	16.3–17.2	17.1	16.6–17.6
Restaurant	Restaurant; pub; steakhouse; cafeteria; typical food restaurant.	16.4	16.0–16.8	21.1	20.5–21.6	11.8	11.4–12.2
Fruit shop	Fruit shop; greengrocer; open air market; *Ceasa*; fruit stand.	1.2	1.1–1.3	1.2	1.1–1.3	1.3	1.2–1.4
Other places	Gas station; convenience store; drugstore, newsstand; hospital; Internet café; public or private company; theater; movies; bookstore; notary public; print shop; particular; third party; sewing store; ignored.	7.7	7.4–8.0	7.6	7.3–8.0	7.8	7.4–8.1

The 567 items purchased for consumption outside the home were grouped into nine categories, considering the nutritional aspects and the marketing characteristics of the item: alcoholic beverage, cookies, sweets, fast food, fruits, milk and dairy products, meals, soft drinks, and snack chips. Those items with reference to more than one type of food in the same meal were placed in their respective groups, i.e., if the subject reported having consumed a sandwich and soft drink, we registered the consumption in the group of fast food and soft drink.

Each subject was classified as a user or not of determined place of purchase and as a consumer or not of a given food group. As the purchase of food could be resumed, we could not estimate how many times a particular place was frequented by the same subject. Thus, we considered a place if the person mentioned at least one purchase there during data collection.

We have estimated the frequency of purchase of food in seven groups of places and according to gender, and we have calculated the average age, income and years of education according to places of purchase of the food consumed outside the home. For the estimation of the type of food purchased at each place among the consumers of a specific place, we used the ratio of the number of subjects who purchased any item in a particular place and all consumers of the place considered. For the estimation of the average *per capita* expenditure in each place, we added all the expenditure incurred and divided it by the total number of subjects per specific place. To calculate the estimates, we considered the sampling weight of the POF and we incorporated the cluster effect of the census sector, using the SAS software, version 9.3.

## RESULTS

The frequency of purchase of food outside the home was reported by 41.2% of the subjects. Men had higher values when compared to women (44%; 95%CI 43.4-44.6 *versus* 38.5%; 95%CI 37.9-39.1). Average age was 34.7 years (95%CI 34.5-34.9), while average years of study was 8.9 years (95%CI 8.8-9.0). Adults had a higher frequency (46%; 95%CI 45.5–46.6) than teenagers (37.7%; 95%CI 36.7–38.6) and older adults (24.2%; 95%CI 23.2–25.2). Average monthly *per capita* household income was R$1,163.21 (95%CI 1,126.91-1,199.51).

The most visited places were snack bar and restaurant, while the fruit shop showed the lowest frequency by both genders. Women showed a higher frequency for the purchase of street food and a lower frequency for restaurants when compared to men (Table 1).

Additional analyses stratified by geographic area and urban or rural situation showed the same order in the choice of places. However, the frequency of purchase of food for consumption outside of the home from supermarkets (5.1% *versus* 3.9%), fruit shops (1.9% *versus* 1.1%) and other places (9.7% *versus* 7.4%) was higher in the rural area than in the urban area. Regarding the macro-regions of Brazil, snack bars and restaurants were the most frequented places in all regions; however, in the North and Northeast, street food had the third highest frequency, in contrast to the other regions, in which other places occupied the third position. In the Midwest, bakeries also appear in third place, along with other places (data not shown).

Subjects who ate in restaurants had higher income, more years of study and were older, while subjects who went to fruit shops had lower income and less years of study (Table 2).

**Table 2:** Socioeconomic and demographic characteristics (95% confidence interval) according to place of purchase of food consumed outside the home. Brazil, 2008-2009.

Place	Age (years)		Level of education (years of study)		Income (R$)	
		
	Average	95%CI	Average	95%CI	Average	95%CI
Supermarket	29.8	29.2–30.3	8.2	7.9–8.5	871.58	797.09–946.07
Bakery	35.0	34.3–35.8	9.3	9.1–9.6	1459.60	1320.05–1599.16
Street food	31.4	30.9–32.0	8.5	8.2–8.8	829.99	765.29–894.69
Snack bar	32.0	31.7–32.3	9.5	9.3–9.6	1193.18	1146.81–1239.55
Restaurant	38.2	37.8–38.5	9.9	9.8–10.0	1656.98	1582.48–1731.48
Fruit shop	32.4	31.1–33.7	7.5	7.1–7.9	678.39	620.34–736.45
Other places	36.2	35.7–36.7	8.3	8.1–8.6	1035.99	972.49–1099.49

Sweets were the most purchased in supermarkets, street food vendors, fruit shops, and bakeries. Snack chips, soft drinks, and fast food were the most purchased in snack bars. The group of snack chips showed the second highest frequency also in fruit shops, bakeries, and street food vendors. At all places, soft drinks were the third most purchased, except in snack bars and fruit shops. In restaurants, meals and alcoholic beverages were mentioned the most (Table 3).

**Table 3:** Frequency of purchase of food groups (95% confidence interval) according to place of purchase of food consumed outside the home. Brazil, 2008-2009.

Place	Alcoholic beverages	Cookies	Sweets	Fast food	Fruits	Milk and dairy products	Meals	Soft drinks	Snack chips
								
	% (95%CI)	% (95%CI)	% (95%CI)	% (95%CI)	% (95%CI)	% (95%CI)	% (95%CI)	% (95%CI)	% (95%CI)
Supermarket	7.1 (6.2–8.0)	22.4 (20.9–23.9)	40.5 (38.7–42.3)	2.8 (2.2–3.5)	3.4 (2.7–4.1)	7.0 (6.1–8.0)	3.3 (2.6–4.1)	17.5 (16.1–18.9)	8.7 (7.6–9.8)
Bakery	1.4 (0.8–2.0)	6.2 (5.2–7.1)	24.8 (22.8–26.7)	11.3 (9.6–13.0)	0.3 (0.1–0.4)	3.3 (2.6–4.1)	1.9 (1.2–2.7)	15.4 (13.6–17.1)	18.0 (16.3–19.7)
Street food	4.0 (3.2–4.8)	3.7 (2.9–4.4)	30.7 (29.0–32.4)	14.8 (13.5–16.1)	3.9 (3.2–4.6)	1.2 (0.9–1.6)	2.3 (1.9–2.8)	16.2 (14.8–17.6)	17.6 (16.3–19.0)
Snack bar	3.7 (3.3–4.2)	2.7 (2.3–3.0)	23.0 (22.1–23.9)	23.4 (22.4–24.4)	0.7 (0.5–0.8)	1.0 (0.8–1.2)	4.2 (3.7–4.6)	24.0 (23.1–25.0)	27.3 (26.3–28.3)
Restaurant	20.4 (19.5–21.2)	0.9 (0.7–1.1)	6.7 (6.1–7.3)	4.5 (4.0–5.0)	0.1 (0.0–0.2)	0.3 (0.2–0.4)	66.7 (65.6–67.8)	16.9 (16.0–17.8)	4.3 (3.9–4.8)
Fruit shop	4.0 (2.9–5.0)	16.1 (13.7–18.4)	26.5 (23.8–29.1)	2.6 (1.6–3.6)	16.5 (13.8–19.2)	3.4 (2.5–4.33)	2.8 (2.0–3.6)	13.8 (11.7–16.0)	21.0 (17.7–24.2)
Other places	6.4 (5.8–7.1)	3.3 (2.8–3.9)	12.1 (11.0–13.2)	4.7 (4.0–5.4)	4.3 (3.8–4.8)	1.85 (1.5–2.2)	49.5 (47.7–51.3)	11.8 (10.6–13.0)	5.3 (4.6–6.1)

The highest spending was recorded among consumers who went to restaurants and other places. Subjects who ate in snack bars spent almost three times less than subjects who chose to eat in restaurants. The places that had, on average, the lowest spending were fruit shops, street food, supermarkets, and bakeries (Table 4).

**Table 4:** Average expenditure on food (95% confidence interval) according to place of purchase of food consumed outside the home in a week. Brazil, 2008-2009.

Place	Expenditure (R$)	

	Average	95%CI
Supermarket	5.43	5.06–5.80
Bakery	6.87	6.31–7.44
Street food	5.05	4.78–5.32
Snack bar	11.54	11.14–11.93
Restaurant	33.24	32.08–34.41
Fruit shop	4.08	3.70–4.45
Other places	20.00	19.25–20.75

## DISCUSSION

The most visited places to purchase food outside the home are snack bars and restaurants. Snack bars combine convenience, practicality, and agility on the food offered, being snack chips, soft drinks, and fast food the most purchased items. These types of food are considered as negative markers of food quality, since they are high in sugar, salt and fat, and low in fiber and micronutrients. These particularities are directly connected to the development of several diseases, including obesity and its comorbidities, as well as the increased risk of nutritional deficiencies, since much of the food is consumed throughout the day and can restrict the consumption of healthy food^14^. In Brazil, the consumption of food outside the home was positively related to the increase in the total energy intake^3^.

On the other hand, restaurants are seen as places that provide healthier food choices, being generally frequented when meals cannot be routinely eaten at home^19^. Restaurants were the second most visited establishment, and meals and alcoholic beverages were the most purchased items. Compared to other places, consumers who went to restaurants had more years of study, higher income, and were older. A study conducted in the city of Campinas, state of São Paulo, Brazil, also showed that restaurant goers have higher level of education and income^17^. Avelar and Rezende^2^ relate increased age with the concern about the quality of the diet.

However, we highlight that food offered by restaurants, in addition to enabling autonomy in food choices and having different types of food, provides a feeling of novelty, being also an option for special occasions. In this study, the percentage of alcohol consumption was higher in restaurants than in other establishments, which can be associated with the celebration of especial events^2^.

Fruits and vegetables are considered as markers of healthy eating as they provide a range of nutrients that protect against various diseases^21^. However, the low purchase of fruits and vegetables is observed not only for consumption outside the home, but also in meals at the home. According to the latest POF of 2008-2009, the household availability of such types of food corresponds to only 2.8% of the total calories, which is considered as insufficient, when we take into account the recommendation for the consumption of 9% to 12% (400 g/day) of total calories for a diet of 2,000 kcal per day^e^.

A population-based study in Brazil, with probabilistic sample of 55,970 households from the POF of 2008-2009, has shown that family income and food prices influence the acquisition of healthy foods, especially among individuals of lower purchasing power^5^. Thus, in order to ensure the caloric contribution in the diet from food with high nutrient density (fruits, vegetables, and milk and dairy products), an increase in the income of the subjects would be needed, or the reduction in prices, as an adequate intake of these types of food creates an increase in expenditure^5^.

In relation to supermarkets, a national-based study conducted in the United States between 2000 and 2006 has found that the greater the distance between the household and the supermarket, the lower the consumption of fruits and vegetables and the higher the prevalence of obesity^11^. Our results show that the purchase of food from supermarkets for consumption outside the home was characterized by products of low nutritional value, such as sweets, cookies, and soft drinks, which can be related to the price, but also with the ease of use and the advertising of offers of such products^16^. The same has been observed by Costa et al.^8^, who have estimated the caloric contribution of food purchased for consumption in the home according to places of purchase in Brazil, identifying supermarkets as the biggest contributors (49%), associated with the consumption of food of low nutritional value, such as ultra-processed food.

In Brazil, we can see a growing participation of processed or ultra-processed products available for consumption^10^. These types of food have a specific profile that promotes excessive consumption of energy, as they can be marketed in greater amounts, have high palatability, have long shelf life, and are easy to transport to the consumer, encouraging the habit of eating them between meals or even replacing them. Another relevant characteristic is the strong marketing for their promotion, based on sales strategies and persuasive ads^12^.

Expenditure with fruit shops, supermarkets, and street food indicates the purchase of fast food or ready meals, as these places generally do not aim to offer physical structure to prepare meals. Meals in restaurants require more preparation time, need cooking skills, and often use more expensive ingredients, which increase the cost of food. Expenditure with places that do not have as their main purpose the sale of food is possibly related with more expensive food items, which justifies the second highest expenditure in the group other places.

Some limitations on the findings of this study should be considered. Information relating the place and type of food purchased for consumption outside the home was obtained by the participation in the total expenditures on food and not from the actual food consumed. This condition hinders the detailed description of the type of food and the amount purchased. In places defined as third party, particular, companies, among others, we could not identify a specific characteristic of the types of products offered or sold, and therefore we considered them in the category other places, what might have directly influenced the high frequency of meals made in that group.

Moreover, another important issue to be considered is that most establishments are not restricted to offer only one type of service and sell different types of food. We can mention supermarkets as an example, which can offer fast food and ready meals, thus hindering the classification of the place of food consumption. The POF of 2008-2009 included a module of individual food consumption; however, despite subjects informing if consumption was made within or outside the home, no detailed information is found on the place (restaurant, snack bar, etc.). This information is available only in the module of individual expenditure, which was used in this study.

As a strength of this study, we highlight the pioneering analysis of the places of purchase of food outside the home based on a representative sample of the Brazilian population, which has allowed us to identify places for the development of public policies that are effective in providing healthy environments that promote adequate diet.

In conclusion, the highest percentage of food consumed outside the home comes from snack bars and restaurants, and the groups of snack chips, soft drinks, and fast food were the most consumed in snack bars and the groups of meals and alcoholic beverages were the most consumed in restaurants. Thus, public policy strategies should be developed in these places, in order to encourage consumers to make healthier choices, focusing on reducing the purchase of processed foods, such as fast food, snack ships, and soft drinks.

## Footnotes

a Instituto Brasileiro de Geografia e Estatística. Pesquisa de Orçamentos Familiares 2008-2009: despesas, rendimentos e condições de vida. Rio de Janeiro; 2010 [cited 2015 Aug 10]. Available from: http://www.ibge.gov.br/home/estatistica/populacao/condicaodevida/pof/2008_2009/POFpublicacao.pdf

b Rodrigues AGM. Estado nutricional, indicadores sociodemográficos, comportamentais e de escolha alimentar de comensais em restaurante de bufê por peso [dissertation]. Florianópolis (SC): Universidade Federal de Santa Catarina; 2011.

c World Health Organization, Division of Food and Nutrition, Food Safety Unit. Essential safety requirements for street-vended foods. rev. ed. Geneva; 1996 [cited 2002 Jun 18]. Available from: http://apps.who.int/iris/bitstream/10665/63265/1/WHO_FNU_FOS_96.7.pdf

d Brasil. Lei nº 11.947, de 16 de junho de 2009. Dispõe sobre o atendimento da alimentação escolar e do Programa Dinheiro Direto na Escola aos alunos da educação básica; altera as Leis nº 10.880, de 9 de junho de 2004, 11.273, de 6 de fevereiro de 2006, nº 11.507, de 20 de julho de 2007; revoga dispositivos da Medida Provisória nº 2.178-36, de 24 de agosto de 2001, e a Lei nº 8.913, de 12 de julho de 1994; e dá outras providências. Brasília (DF); 2009 [cited 2016 Dec 5]. Available from: http://www.planalto.gov.br/ccivil_03/_ato2007-2010/2009/lei/l11947.htm

e Instituto Brasileiro de Geografia e Estatística. Pesquisa de Orçamentos Familiares 2008-2009: avaliação nutricional da disponibilidade domiciliar de alimentos no Brasil. Rio de Janeiro; 2010 [cited 2015 Aug 10]. Available from: http://www.ibge.gov.br/english/estatistica/populacao/condicaodevida/pof/2008_2009_aval_nutricional/pof20082009_avaliacao.pdf
